# Visual Performance, Satisfaction, and Spectacle Independence after Implantation of a New Hydrophobic Trifocal Intraocular Lens

**DOI:** 10.3390/jcm11195931

**Published:** 2022-10-08

**Authors:** Antonio Cano-Ortiz, Álvaro Sánchez-Ventosa, Timoteo González-Cruces, David Cerdán-Palacios, Vanesa Díaz-Mesa, Rubén Gallego-Ordóñez, Teresa Gálvez-Gómez, Jose A. García Parrizas, Javier Zurera Baena, Alberto Villarrubia-Cuadrado

**Affiliations:** Department of Ophthalmology, Hospital La Arruzafa, 14012 Córdoba, Spain

**Keywords:** trifocal intraocular lens, visual questionnaire, spectacle independence

## Abstract

The main objective was to evaluate distance, intermediate, and near vision in patients who have undergone cataract extraction with bilateral implantation of a new trifocal diffractive intraocular lens (IOL), along with patient-reported outcomes (PRO). A total of 50 eyes from 25 patients after Asqelio^TM^ Trifocal IOL (AST Products, Inc., Billerica MA, USA) implantation were assessed in this study. At 3 months after surgery, the photopic visual acuity (VA) at distance, intermediate, and near distances was measured. Binocular photopic defocus curves were also obtained. Three questionnaires to assess patients’ visual satisfaction and spectacle dependence, among other items, were completed: the Catquest-9SF, the patient-reported spectacle independence questionnaire (PRSIQ), and the patient-reported visual symptoms questionnaire (PRVSQ). The average spherical equivalent was 0.21 ± 0.37 D at 3 months post-operation, and the average absolute tolerance to defocus was 3.64 ± 0.70 D. The mean binocular uncorrected VAs for distance, intermediate, and near vision were −0.02 ± 0.09, 0.06 ± 0.08, and 0.11 ± 0.07 logMAR, respectively. The best-corrected VA was better than 0.1 logMAR for the whole range from distance to near. PROs revealed spectacle independence and general satisfaction with vision, and the incidence of photic phenomena were low. This study shows that the new bi-aspheric diffractive trifocal IOL provides a good visual performance at different distances under photopic conditions, accompanied by patient satisfaction and spectacle independence.

## 1. Introduction

Trifocal intraocular lenses (IOLs) have been designed in order to provide patients with an adequate visual acuity at different distances, from far to near. Different designs have been launched on the market and are available for consideration by cataract and refractive surgeons. Some reviews have shown that the use of trifocal IOLs in cataract patients improves intermediate visual acuity compared to patients implanted with bifocal IOLs [1,2,3]. The addition of a third focus, specifically designed to enhance intermediate vision, compared to classic bifocal designs has increased the use of trifocal IOLs among surgeons aiming to provide their patients with a better visual performance over the entire range of distances.

The visual performance of patients submitted for cataract surgery depends on the IOL type considered. The limited depth of focus of bifocal IOLs means that they cannot provide clear and useful vision at intermediate distances. This was the primary reason for the development of trifocal IOLs: to provide pseudophakic patients with the possibility of useful vision across a wide range, thus allowing a reduction in spectacle dependence. Despite the improvement in visual acuity observed with trifocal IOLs, some complaints of glare and/or halos may be found in these types of designs [3,4]. There are different designs of trifocal IOLs created by IOL manufacturers and, therefore, a different design may provide different visual outcomes when implanted. The outcomes of any trifocal IOL should be analyzed properly, specifically focusing on visual performance at all distances, but also considering the patient’s satisfaction and spectacle dependence as we have previously indicated.

One new trifocal IOL currently available on the market is the Asqelio^TM^ trifocal IOL (AST Products, Inc., Billerica, MA, USA). This lens has a bi-aspheric geometry, with a posterior diffractive optic. The purpose of the present clinical study was to study the refractive and visual outcomes of patients implanted with the Asqelio^TM^ trifocal IOL. Besides visual performance, the study also included patient-reported outcomes to assess spectacle independence, satisfaction with visual outcomes at different distances, and the incidence of visual symptoms such as glare and/or halos.

## 2. Materials and Methods

This was a retrospective study involving patients bilaterally implanted with the Asqelio^TM^ non-toric trifocal IOL. This IOL was implanted following cataract extraction with phacoemulsification, and targeted for emmetropia. The inclusion criteria for enrollment included patients of 40 years of age or more who want to be spectacle independent at any distance, no active ocular disease except for cataract, non-severe dry eye, uneventful cataract surgery and post-operative healing process, clear posterior capsule and lens implant, no pupillary abnormality, and the capacity to read and understand questionnaires. The exclusion criteria were irregular corneal astigmatism, extreme pupil size (photopic value <3.00 mm and/or scotopic value >7.00 mm), previous corneal or intraocular refractive surgery, corneal anomalies, IOL dislocation, posterior capsule opacification, corneal astigmatism >1.00 diopter (D), or any vitreous or retinal disease. The first 25 consecutive patients bilaterally implanted with spherical non-toric Asqelio^TM^ trifocal IOLs at the La Arruzafa Hospital (Córdoba, Spain) who met the inclusion criteria and agreed to complete the visual outcomes questionnaires were recruited.

In agreement with the Declaration of Helsinki, the protocol of the study was reviewed and approved by the Reina Sofia University Hospital Ethics Committee.

### 2.1. Intraocular Lens

**Figure 1:**
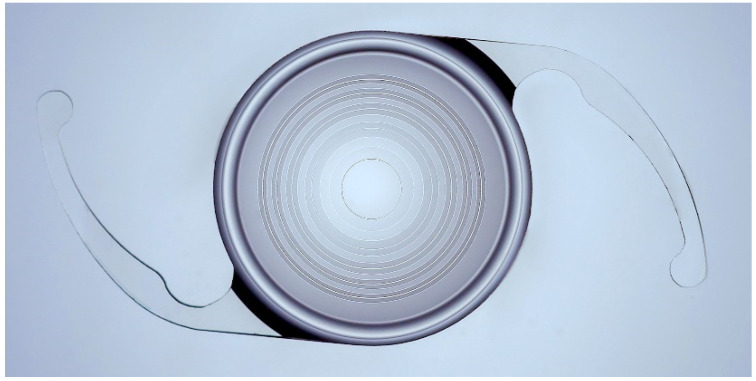
Asqelio^TM^ Trifocal TFLIO130C.

All patients were implanted with the Asqelio^TM^ trifocal TFLIO130C model IOL (AST Products, Inc., Billerica, MA, USA). This IOL has a bi-aspheric geometry with a posterior diffractive optic design (15 rings within the central 4.5 mm) in its 6.0 mm-diameter optical zone (Figure 1). It has a total diameter of 13.0 mm and provides an addition for near distances of +3.30 D and +2.20 D for intermediate distances. The lens is built in powers ranging from +5.00 to +34.00 D in 0.50 D increments, with a C-Loop platform and a light distribution among its foci of 50% for distance, 24% for intermediate, and 26% for near. It is made of a soft hydrophobic acrylic material (glistening-free) with a refractive index of 1.50, Abbe number of 50, and spherical aberration of −0.27 microns.

### 2.2. Clinical Procedures

Prior to surgery, patients underwent a comprehensive ophthalmological examination including optical biometry and anterior surface optical tomography in order to calculate the power of the IOL.

Biometry was determined using the IOLMaster 700 (Carl Zeiss Meditech, Jena, Germany). The device uses swept-source OCT technology with a wavelength of 1050 nm to determine axial length, anterior chamber depth, and both corneal and lens thicknesses.

IOL calculation was carried out using the biometric parameters provided by the IOLMaster and applying the Barrett II universal formula. In all cases, the IOL power chosen was the one yielding myopic values closest to zero. No restrictions were applied with regards to axial length.

### 2.3. Surgical Procedure

Surgical procedures with IOL implantation were conducted with a difference of 7 days on average between eyes. All patients were submitted for cataract surgery by phacoemulsification according to regular clinical practice procedures. Surgical procedures were conducted by the same experienced surgeon (A.C.O.) under local anesthesia through a micro-incision of 2.2 mm.

### 2.4. Post-Operative Assessment

Patients were examined after IOL implantation according to the usual post-operative follow-up visits. During these visits, the visual and ocular status of the patient were revised.

Visual acuity (VA) was determined with and without the best compensation for distance. Post-operatively, measurements were carried out uncorrected for distance, and best-distance-corrected for far distance using a retro-illuminated ETDRS vision chart (Precision Vision, Woodstock, NY, USA) at 85 cd/m², and intermediate and near distances using logarithmic visual acuity charts, (scrambled lines from the ETDRS original and ETDRS 2000 series), calibrated for testing at 40 (for near) and 66 (for intermediate) cm, with size increments = 0.1 log units (Precision Vision, Woodstock, NY, USA).

Defocus curves were obtained binocularly from all participants under photopic conditions at 3 months post-operation. The step size in diopters was 0.50 D, ranging from +2.00 to −4.00 D. VA was measured in the logMAR scale, and the optotype used was the ETDRS at 4 m. All participants were measured with the best correction for distance vision to compensate for residual refractive errors.

The subjective tolerance to defocus was determined from defocus curves. The VA criteria to determine the threshold that defines the defocus tolerance is of the highest importance; commonly, 0.1 logMAR is used. An absolute criterion was applied to obtain the defocus tolerance range from those vergences (in D) which provided VA values ≤0.1 logMAR, as previously used by some authors [5,6].

### 2.5. Questionnaires

Patients were asked to complete three questionnaires: the Spanish version of the Catquest-9SF patient outcomes questionnaire, the patient-reported spectacle independence questionnaire (PRSIQ), and the patient-reported visual symptoms questionnaire (PRVSQ). The Catquest-9SF is a 9-item questionnaire to determine patient activity limitations in daily life due to poor vision, and is used due to its documented responsiveness in cataract surgery [7,8]. It has been translated into many languages, validated through Rasch analysis [9], and is recommended by the International Consortium of Health Outcomes Measurement [10] and the European Registry of Quality Outcomes for Cataract and Refractive Surgery [11].

It comprises 9 items with 4 response options, ranging from 4 for “very great difficulty/very dissatisfied” to 1 for “no difficulty/very satisfied”, and a “cannot decide” additional option, which is treated as missing data. Items A and C1–C7 are concerned with difficulty, while item B deals with satisfaction. The PRSIQ [12] is used to determine subjects’ need for spectacles or contact lenses and their satisfaction with vision at various distances. It comprises 13 items with 5 response options, ranging from 5 for “never/ completely satisfied” to 1 for “all the time/dissatisfied”. Items 1a to 1d are concerned with spectacle independence (use of additional correction) at the different distances, items 2a to 2d assess satisfaction with vision without additional correction of those differences, and items 3a to 3e assess satisfaction with performance while carrying out different visual tasks. The PRVSQ is used to determine the presence and extent of visual symptoms such as halos, starbursts, and glare. It comprises 6 items with 5 response options. Items 1a, 2a, and 3a relate to the incidence of halos, starbursts, and glare, respectively, and responses range from “never” to “all the time”. Items 1b, 2b, and 3b assess the level of impairment caused by these disturbances, and responses range from “not at all” to “extremely”.

### 2.6. Statistical Analysis

Statistical analysis of the results was carried out using IBM^®^ SPSS^®^ for Mac v.26.0.0 (SPSS Inc., Chicago, IL, USA). Paired samples t-tests were used to assess differences in the refractive error components. Correlation analyses were used to determine agreement between the responses to satisfaction items between questionnaires. All statistical tests were 2-tailed, and *p*-values lower than 0.05 were considered statistically significant. Outcomes from the questionnaires were analyzed as frequencies and percentages.

## 3. Results

A total of 25 cataract surgery patients binocularly implanted with the Asqelio^TM^ Trifocal IOL (AST Products, Inc., Billerica, MA, USA) were enrolled in the study (15 female and 10 male; mean age 63.6 ± 9.7 years, ranging from 44 to 80). The descriptive statistics of the sample are displayed in Table 1.

### 3.1. Post-Operative Refraction

**Figure 2:**
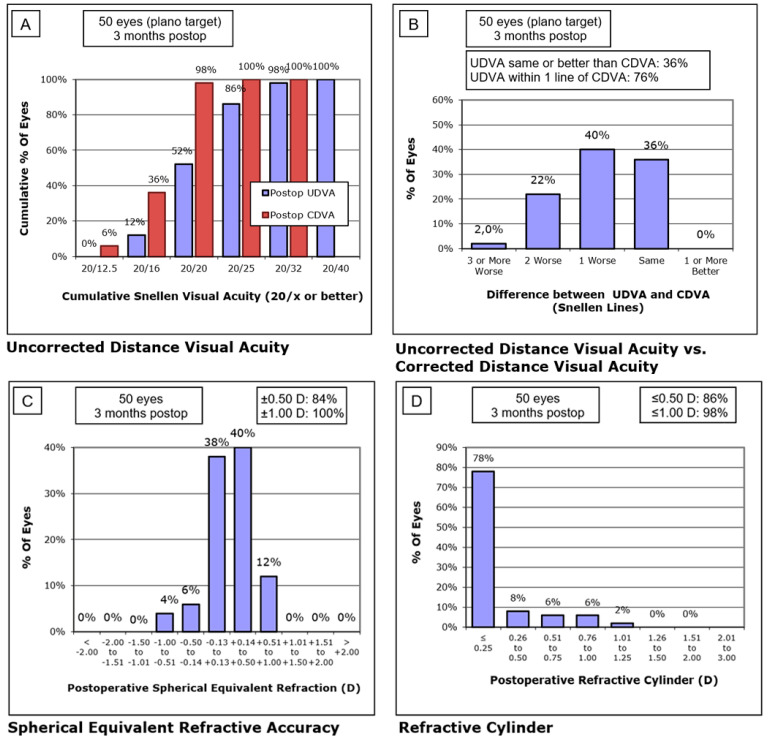
Refractive and visual postoperative outcomes. (**A**) Cumulative Snellen uncorrected distance visual acuity; (**B**) Difference between uncorrected distance visual acuity versus corrected distance visual acuity; (**C**) Postoperative spherical equivalent refraction; (**D**) Postoperative residual refractive cylinder.

**Figure 3:**
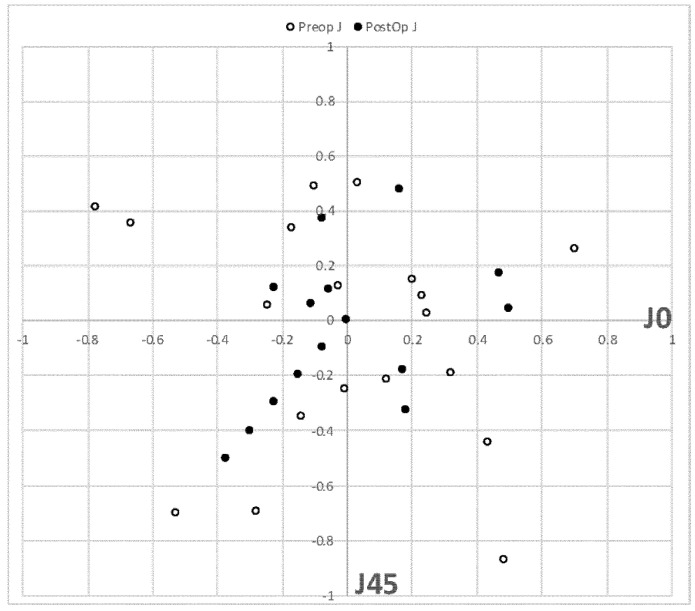
Distribution of residual astigmatism (D) scatterplot (J0 vs. J45). Empty bins represent preoperative data, and full black bins 3 months postoperation.

The average spherical equivalent was 0.21 ± 0.37 D at 3 months post-operation (range from −0.87 to +1.00 D). The average residual cylinder was −0.20 ± 0.35 D at 3 months post-operation. Figure 2 provides the post-operative standard refractive graphs. Regarding refractive astigmatic outcomes, paired-samples t-tests of the astigmatic components J0 and J45 did not reveal statistically significant differences pre- and post-operatively (*p* > 0.05). Figure 3 displays the distributions of pre-operative astigmatism and residual post-operative astigmatism.

### 3.2. Visual Performance

**Figure 4:**
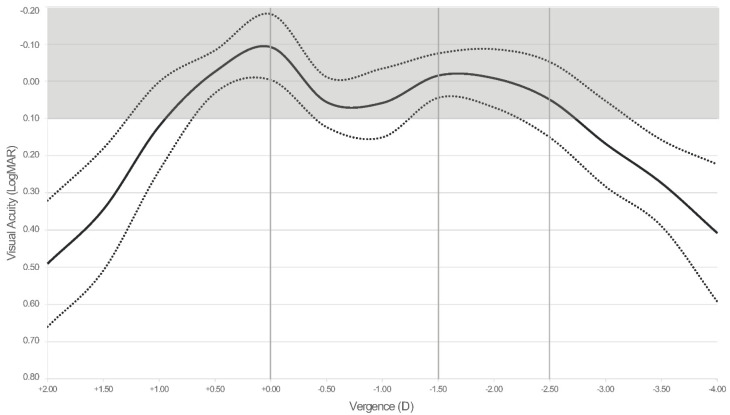
Average defocus curve 3 months after intraocular lens implantation (*n* = 50 eyes, 25 patients). Dotted curves represent the ± standard deviation curves. For reference purposes, dashed vertical grey lines highlight the location of far, intermediate (67 cm), and near vision (40 cm) on the vergence axis, and the grey shadowed area highlights that area on the chart corresponding to visual acuity values above 0.1 logMAR (equivalent to 1.0 Snellen decimal or 20/20).

Table 2 displays the average visual acuities obtained for far, intermediate, and near distances pre-operatively and 3 months after surgery. Figure 4 shows the average binocular defocus curve 3 months after IOL implantation. The average visual performance across the defocus curve does not drop below 0.1 logMAR at any point from +1.00 D to −2.75 D, yielding an average absolute tolerance to defocus of 3.64 ± 0.70 D.

### 3.3. Outcomes of the Questionnaires

All patients reported spectacle independence for distance vision, while 96% (24) also did so for intermediate vision, 88% (22) for near vision, and 92% (23) reported independence for vision in general (at all distances). Spectacle independence for intermediate vision was positively correlated with independence for near vision (Spearman’s rho 0.602, *p* = 0.001) and with independence to all distances (Spearman’s rho 0.692, *p* < 0.001). Spectacle independence for intermediate and near vision was also positively correlated to finding difficulties performing tasks such as looking at prices when shopping, doing handicrafts, or engaging in hobbies (*p* < 0.05 in all cases).

More than 80% (20) of patients reported being very satisfied or completely satisfied with their distance, intermediate, or general vision without additional compensation (spectacles or contact lenses). This percentage was reduced to 56% (14) who were very satisfied or completely satisfied with their near vision, and only 1 patient reported being unsatisfied with his near vision. Table 3 displays the responses in spectacle independence and satisfaction as per the PRSIQ.

All patients reported being fairly satisfied (scoring 3) to very satisfied (scoring 4) with their vision at present (mean score 3.60 ± 0.50), and 20% (5) reported finding some difficulties with their vision in their daily life, while 80% (20) did not report any difficulty (mean score 3.80 ± 0.41). With regards to difficulties carrying out different tasks, in general between 12% (3) and 24% (6) of patients reported some difficulty performing the tasks defined in the Catquest-9SF, while most of the patients did not. Table 4 summarizes the patient-reported difficulties with their vision as per the Catquest-9SF.

**Figure 5:**
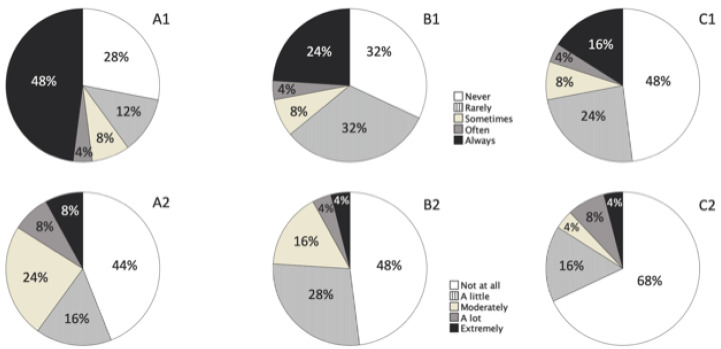
Pie charts showing the distribution of reported visual experience within the last 7 days (upper row) and level of impairment (lower row) of halos (**A1**,**A2**), starbursts (**B1**,**B2**), and glare (**C1**,**C2**), as per the PRVSQ. (**A1**) percentage of halos symptoms; (**A2**) subjective level of halos; (**B1**) percentage of starbursts symptoms; (**B2**) subjective level of starbursts; (**C1**) percentage of glare symptoms; (**C2**) subjective level of glare. The legends in the middle apply to all the pie charts within each row, respectively.

More than half of the patients experienced halos often or all the time, but only 16% (4) reported a lot of/extreme impairment as a result. With regards to starbursts, 28% (7) reported experiencing them often or all the time, but only 8% (2) reported a lot of/extreme impairment. In the case of glare, 20% (5) reported experiencing this often or all the time, and 12% (3) reported a lot of/extreme impairment. Impairment as a result of glare reported by patients was correlated with satisfaction with spectacle independence at all distances (0.581, *p* = 0.002), as well as satisfaction performing tasks without additional correction, such as seeing objects or signs at sunset or at night (0.576, *p* = 0.003), seeing borders or steps at sunset or at night (0.631. *p* = 0.001), or seeing numbers or indicators on the car dashboard (0.531, *p* = 0.011). Figure 5 shows pie charts displaying the percentages of visual symptoms experienced by the patients and the level of impairment as per the PRVSQ.

## 4. Discussion

It has been reported in different clinical studies that trifocal IOLs can improve distance and near vision while providing a good level of intermediate vision. This type of IOL has been reported to produce less limitation in visual function and less spectacle dependence than seen in patients implanted with bifocal or monofocal IOLs [1,2,3].

The present study, at 3 months post-surgery, supports the good refractive outcomes by obtaining both spherical equivalent and cylinder mean values below a quarter of diopter. With regards to visual acuity outcomes, at distance, CDVA was >20/20 for both monocular and binocular viewing conditions. These values were slightly worse for intermediate and near vision but, in any case, always ≥20/25. The lens seems to provide better results for distance vision while providing good outcomes for intermediate and near vision as well. In addition, the good subjective perception of patients for intermediate and near vision suggests the correct behavior of the IOL for those distances. Regarding the defocus curve, there is a change in visual acuity as a function of the vergence (distance). The curve shows a peak at 0 D of vergence (distance vision) with a mean visual acuity about 20/16, followed by a smooth valley up to −1.50 D of vergence and another peak at about −1.75 D (equivalent to approximately 60 cm) with a visual acuity >20/20. Note that considering the values reported for the whole curve, the mean visual performance does not drop below 0.1 logMAR at any point from +1.00 D to −2.75 D, yielding a mean absolute tolerance to defocus of 3.64 ± 0.70 D. In a similar study, using another trifocal IOL model, Poyales et al. [13] found a visual acuity of 0.3 logMAR or better in the range from 0.5 to −2.5 D of vergence. This value compares well with the better performing lenses in previous studies of tolerance to defocus after implantation of different multifocal IOLs in which the 0.1 logMAR criterion was applied, as used in the present study [14,15].

It must be considered that the analysis of patient-reported outcomes in the present study is limited by the study sample size. Each response has a significant impact on the overall percentage; therefore, the patient-reported outcomes obtained here must be considered as trends that would need confirmation over a greater sample.

The Spanish version of the Catquest-9SF questionnaire used has been previously validated for patient-reported visual function with trifocal IOLs, showing very good psychometric properties [16]. In a previous study on patient-reported outcomes using a different diffractive trifocal IOL design in which the Catquest-9SF was used [17], no-one responded that they had great or very great difficulties, the same results obtained in the present study. Additionally, 100% of the individuals surveyed manifested that they felt satisfied with their vision, similar to those found in the present study (60% very satisfied and 40% fairly satisfied). Regarding near vision, 56% of patient responded as very satisfied or completely satisfied, despite excellent results obtained in objective visual tests. This lack of correlation between objective and subjective tests has also been found in similar studies. The authors believe that it is due to the various contrasts and lighting conditions that patients experience in real life. None of the patients answered that they were rather or very dissatisfied with the results in their study, as occurred in the present study. Rementería-Capelo et al. [18] also used the Spanish Catquest-9SF to evaluate patient-reported visual function with a diffractive trifocal IOL, in both spherical and toric versions. Although their percentages were not provided, they reported that patients in both groups said they had little difficulty in their everyday life, without statistically significant differences between the toric and spherical designs. Overall satisfaction with post-operative vision was also good in both groups.

Patient-reported spectacle independence was assessed using a non-validated Spanish translation of the English validated PRSIQ [12]. In a study assessing patient-reported spectacle independence 6 years after implantation of a trifocal IOL, Fernandez et al. [19] used the same questionnaire and reported the total spectacle independence for far and intermediate distance vision, but 19.4% of subjects reported a need for spectacle use for near vision, 6.5% reported this aid was sometimes needed, and 12.9% rarely needed it. Song et al. [20] reported good visual outcomes by combining a trifocal and an EDOF IOL using the Visual Function Questionnaire (VFQ-25). Only 4% of patients needed spectacles at the near distance. In the present study, only 1 subject reported seldom needing spectacles for intermediate distances, while for near vision, 88% (22) achieved spectacle independence, and the remaining 12% (3) reported a need for spectacles rarely (1 patient), sometimes (1 patient), and all of the time (1 patient). In the present study, 100% of subjects reported being satisfied or very satisfied with their vision at all distances (PRSIQ, question 2 D), the same as reported by Vargas et al. [21] (100%), and higher than reported by Fernandez et al. [19] and Liu et al. [22] (more than 90%), and Mendicute et al. [23] (more than 80%).

With regards to visual symptoms, the most frequent concerns with multifocal correction of presbyopia relate to blurred vision and the presence of positive dysphotopsias, such as glare, halos, and starbursts [24]. In the present study, the most prevalent photic phenomenon reported by patients was the halo, more than half of the patients (52%, 13 patients) reported perceiving it all the time, but it was not significantly bothersome for 84% (21) of patients, and related to reported satisfaction performing tasks at sunset or at night. However, the presence of halos may be more evident in extreme lighting conditions such as during night driving. The light splitting inherent to multifocal IOL designs results in increased scattered light and the generation of multiple out-of-focus images on the retina. Halo formation results from the superimposition of these out-of-focus images on the focused image projected on the retina [25]. Teshigwara et al. [26] showed that the change in halo size and intensity of a diffractive trifocal and a hybrid multifocal-EDOF IOLs over time should be considered during treatment planning, and they suggest that age, target refraction, and pre-operative pupil size might be predictive factors for halo size. Rementería-Capelo et al. [27] reported that the mean halo effect after diffractive trifocal implantation was lower when no residual refractive error is induced, with a higher halo perception with defocus, being similar both for negative and positive defocus. In a recent review of methods for determining the impact of photic phenomena in patients implanted with trifocal and EDOF IOLs, including patient-reported outcomes questionnaires [28], a need for the standardization of patient-reported outcomes in clinical research was highlighted. One limitation of the present study is the luck of the objective visual results in mesopic conditions.

To summarize, the new bi-aspheric hydrophobic diffractive trifocal IOL provides a good visual performance at different distances under photopic conditions, with a tolerance to defocus within the range from distance to near vision above 0.1 logMAR (0.8 decimal Snellen). The three questionnaires used in this study show spectacle independence and patient satisfaction after implantation, although these patient-reported outcomes must be confirmed in larger samples. Visual symptoms, particularly halos, are prevalent as expected given the multifocal character of lens design, but not significantly bothersome for the patients. Future studies with larger sample sizes and longer follow-ups should be conducted to confirm these findings.

## Figures and Tables

**Table 1:** Descriptive statistics of the sample.

	Min	Max	Mean ± SD
Pre-Operative SE (D)	−4.25	7.38	1.52 ± 2.14
Pre-Operative J0 (D)	−0.77	0.70	−0.01 ± 0.31
Pre-Operative J45 (D)	−0.87	0.50	−0.01 ± 0.28
AL (mm)	20.37	27.02	22.92 ± 1.28
ACD (mm)	2.31	3.67	3.05 ± 0.32
K1 (D)	39.14	47.10	43.67 ± 1.65
K2 (D)	39.41	47.95	44.19 ± 1.64
IOL Power (D)	17.00	32.00	23.29 ± 3.22

SE: spherical equivalent; AL: axial length; ACD: anterior chamber depth; K1: flat corneal meridian; K2: steep corneal meridian; IOL: intraocular lens; SD: standard deviation.

**Table 2:** Monocular visual acuity obtained for far, intermediate, and near distances pre-operatively and three months after surgery. All values are in logMAR units.

	Min	Max	Mean	SD
Monocular UDVA	−0.10	0.30	0.05	0.09
Monocular CDVA	−0.22	0.02	−0.04	0.06
Monocular UIVA	0.00	0.20	0.07	0.08
Monocular DCIVA	0.00	0.30	0.08	0.10
Monocular UNVA	0.00	0.30	0.14	0.07
Monocular DCNVA	0.00	0.20	0.10	0.08
Binocular UDVA	−0.22	0.20	−0.02	0.09
Binocular CDVA	−0.22	0.00	−0.06	0.06
Binocular UIVA	0.00	0.20	0.06	0.08
Binocular DCIVA	−0.10	0.28	0.02	0.09
Binocular UNVA	0.00	0.30	0.11	0.07
Binocular DCNVA	0.00	0.20	0.06	0.07

UDVA: uncorrected distance visual acuity; CDVA: corrected distance visual acuity; UIVA: uncorrected intermediate visual acuity; DCIVA: distance-corrected intermediate visual acuity; UNVA: uncorrected near vision acuity; DCNVA: distance-corrected near visual acuity; SD: standard deviation.

**Table 3:** Summary of 25 patients’ reported outcomes for spectacle independence as per the PRSIQ. Response coding for items 1A–1E/2A–3E: R1 (all the time/completely satisfied), R2 (most of the time/very satisfied), R3 (sometimes/fairly satisfied), R4 (rarely/little satisfied), R5 (never/unsatisfied). SD: standard deviation.

	Mean ± SD	Mode	Response Frequencies (%)				
			R1	R2	R3	R4	R5
1. Within the last 7 days, how often did you wear glasses (including reading glasses) or CL?							
A. To see at far distance (1.5 m or more)	5.00 ± 0.00	5	0	0	0	0	100
B. To see at intermediate distance (0.5–1.5 m)	4.96 ± 0.20	5	0	0	0	4	96
C. To see at near distance (less than 0.5 m)	4.72 ± 0.89	5	4	0	4	4	88
D. To see in general (all distances)	4.84 ± 0.55	5	0	0	8	0	92
2. What is your level of satisfaction without glasses (including reading glasses) or CL?							
A. To see at far distance (1.5 m or more)	1.68 ± 0.63	2	40	52	8	0	0
B. To see at intermediate distance (0.5–1.5 m)?	1.80 ± 0.76	1	40	40	20	0	0
C. To see at near distance (less than 0.5 m)?	2.32 ± 1.18	1	32	24	28	12	4
D. To see in general (all distances)?	1.72 ± 0.61	2	36	56	8	0	0
3. Generally, what is your level of satisfaction when performing these tasks?							
A. Reading a menu in a restaurant with dimmed light	2.28 ± 1.14	1	32	24	32	8	4
B. Seeing objects or reading signs on the streets at sunset or at night	2.16 ± 0.90	2	24	44	24	8	0
C. Seeing steps or borders at sunset or at night	1.88 ± 0.78	2	36	40	24	0	0
D. Looking at photos on a smartphone or tablet	1.84 ± 0.94	1	44	36	12	8	0
E. Reading numbers and indicators from the car dashboard	1.82 ± 0.79	1	36	32	20	0	0

**Table 4:** Summary of 25 patients’ reported difficulties with their vision as per the Catquest-9SF. Response coding: R1 (yes, very great difficulty), R2 (yes, great difficulty), R3 (yes, some difficulty), R4 (no, no difficulty), R5 (cannot decide). SD: standard deviation.

	Mean ± SD	Mode	Response Frequencies (%)				
			R1	R2	R3	R4	R5
Do you find that your sight at present in some way causes you difficulty in your everyday life?	3.80 ± 0.41	4	0	0	20	80	0
Do you have difficulty…							
…Reading text in newspapers?	3.84 ± 0.47	4	0	0	20	76	4
…Recognizing the faces of people you meet?	3.80 ± 0.41	4	0	0	20	80	0
…Seeing the prices of goods when shopping?	3.76 ± 0.52	4	0	4	16	80	0
…Seeing to walk on uneven surfaces?	3.88 ± 0.33	4	0	0	12	88	0
…Seeing to do handicrafts, woodwork, etc.?	3.76 ± 0.66	4	0	4	24	64	8
…Reading subtitles on TV?	3.88 ± 0.33	4	0	0	12	88	0
…Seeing to engage in an activity/hobby?	3.76 ± 0.52	4	0	4	16	80	0

## Data Availability

The data that support the findings of this research are available from the corresponding author upon reasonable request.
